# Effects of COVID-19 Misinformation on Information Seeking, Avoidance, and Processing: A Multicountry Comparative Study

**DOI:** 10.1177/1075547020959670

**Published:** 2020-10

**Authors:** Hye Kyung Kim, Jisoo Ahn, Lucy Atkinson, Lee Ann Kahlor

**Affiliations:** 1 Nanyang Technological University, Singapore; 2 Hallym University, Chuncheon, Republic of Korea; 3 The University of Texas at Austin, Austin, TX, USA

**Keywords:** COVID-19, cross-country comparison, information seeking and processing, misinformation

## Abstract

We examined the implications of exposure to misinformation about COVID-19 in the United States, South Korea, and Singapore in the early stages of the global pandemic. The online survey results showed that misinformation exposure reduced information insufficiency, which subsequently led to greater information avoidance and heuristic processing, as well as less systematic processing of COVID-19 information. Indirect effects differ by country and were stronger in the U.S. sample than in the Singapore sample. This study highlights negative consequences of misinformation during a global pandemic and addresses possible cultural and situational differences in how people interpret and respond to misinformation.

The pandemic caused by the coronavirus disease 2019 (hereafter referred to as COVID-19) poses unprecedented threats to global human well-being. Because of the high uncertainty associated with the novelty of COVID-19, many people rely on online health information to learn more about how to protect themselves and their families from the imminent health threat (Bento et al., 2020; Garfin et al., 2020; Hernández-García & Giménez-Júlvez, 2020). While its prevention and treatment require practices based on scientific evidence, there are myriad sources of incorrect information circulating on the internet about what prevents and cures COVID-19. This is critical because relying on such misinformation can bring about detrimental health outcomes by encouraging people to engage in ineffective—even harmful—remedies.

For example, nearly 300 people have been killed by ingesting methanol based on harmful treatment recommendations that spread across social media in Iran (Associated Press, 2020). In South Korea, 46 churchgoers were infected with COVID-19 after church leaders sprayed saltwater into their mouths out of a misguided belief that the water would help prevent the spread of COVID-19; the spray bottle became contaminated with the virus in the process and spread infection (Park, 2020). In the United States, rumors spread on social and national media that ingesting bleach might help kill the virus; research suggests that this misinformation contributed to individuals “engaging in non-recommended high-risk practices with the intent of preventing SARS-CoV-2 transmission, such as washing food products with bleach, applying household cleaning or disinfectant products to bare skin, and intentionally inhaling or ingesting these products” (Gharpure et al., 2020, p. 705). Accordingly, the World Health Organization (WHO; 2020) has declared an “infodemic” related to COVID-19 and actively sought to rectify the crisis levels of misinformation spreading online.

Despite the proliferation of online misinformation, the internet is an important source of information during a disease pandemic as it can be an efficient and expeditious channel for providing necessary information and for correcting misinformation. Indeed, risk communication scholars have emphasized the importance of providing timely information in risk contexts to help aid decision making, especially when there is considerable uncertainty about the most effective course of action in a given situation (Edgar et al., 2000; Yang, Aloe, & Feeley, 2014). Therefore, scholars must find ways to better understand how the power of the Internet to *misinform* is affecting its ability to *inform*. One important, still unanswered question is whether exposure to misinformation serves to motivate or deter subsequent information seeking, and/or changes the way encountered information is processed. Prior theorizing on risk communication has often focused on immediate outcomes of information exposure; yet there is a lack of understanding about the subsequent information management that follows exposure to risk information (So et al., 2019). The current study addresses these important gaps in the extant literature in its examination of exposure to misinformation on COVID-19.

This study builds on previous research by offering two main contributions. First, guided by the risk information seeking and processing (RISP) model (Griffin et al., 1999), we posit that a reduction in the perceived need for additional information (or *information insufficiency*) is an important mechanism that underlies adverse consequences of misinformation exposure on subsequent information seeking and processing. Given that prior research has often focused on the spread of misinformation (Guess et al., 2019; Valenzuela et al., 2019), addressing the implications of that spread on information seeking and processing helps to enrich our understanding of misinformation effects. Second, this current study examines whether the effects of misinformation exposure are universal across cultures or specific to certain cultural contexts. Studies on information seeking and processing have been conducted predominantly in Western contexts, and cross-cultural studies, especially studies that involve multiple countries, are lacking in the extant literature. For theory building and refinement, it is important to examine the validity of a theoretical prediction across different cultural contexts and populations.

## The Spread of Misinformation During a Pandemic

Misinformation is defined as objectively incorrect information that is not supported by scientific evidence and expert opinion (Nyhan & Reifler, 2010). While misinformation can persist for a long time without contradiction (Kata, 2010), for scientific issues, what is true or false can be altered with newly emerging evidence and consensus among experts (Vraga & Bode, 2020). Researchers also differentiate misinformation from misperception and disinformation: Misperception is holding a belief that is incorrect or false (Southwell et al., 2018), whereas disinformation is driven by the intention to deceive (Wardle, 2017). While misinformation is inadvertently false, its propagation or sharing can subsequently be either deliberative or accidental (Southwell et al., 2018).

There is ample evidence on the pervasiveness of misinformation in the context of infectious disease outbreaks. For example, a study on the Zika virus found that half of the top 10 news stories were based on misinformation or rumors, and those stories were 3 times more likely to be shared on social media than stories based on facts (Sommariva et al., 2018). Studies on COVID-19 similarly found that misinformation was more frequently tweeted than science-based evidence or public health recommendations (Bridgman et al., 2020; Pulido et al., 2020). Researchers have addressed the potential consequences of misinformation that could undermine the adoption of preventive measures (Bridgman et al., 2020; Dixon & Clarke, 2012; Tan et al., 2015), which could exacerbate the spread of the epidemic.

Researchers have also suggested that exposure to misinformation can trigger individuals’ additional information seeking to verify the information that they suspect to be false (Tandoc et al., 2017). For example, when individuals cannot verify information on social media based on their own judgment and knowledge, they seek out information from their social circle and other sources to authenticate (Tandoc et al., 2017). Researchers, however, also point out that the motivation for subsequent information seeking may not always be related to accuracy (Southwell, 2013). As Thorson (2016) addressed, misinformation exposure actually may prevent individuals from seeking new information and instead may trigger motivated processing to protect their preexisting attitudes or beliefs. Such selective information exposure and motivated reasoning make it difficult to rectify misinformation once false beliefs are deeply held (Bode & Vraga, 2015; Jerit & Barabas, 2012). As Porter and Wood (2019) argued, however, individuals can simultaneously pursue the accuracy on factual matters as well as the goals that serve their pre-extant beliefs.

In the context of a novel disease pandemic, it is also important to consider the uncertainty regarding what is true and false about the disease and its prevention, given that the expert consensus and “best available evidence” are subject to change (Vraga & Bode, 2020, p. 138). Such information uncertainty may as well have implications on the public’s information behaviors. In a recent study, perceived exposure to COVID-19 misinformation was positively associated with seeking more information and complying with health advisories (Hameleers et al., 2020). Because *perceived* misinformation exposure takes into account individuals’ judgment on the veracity of information, its implications may differ from exposure to misinformation that is based on actual state of scientific evidence (Vraga & Bode, 2020). We thus take the latter conceptualization in the current study to understand the effects of misinformation exposure.

## Information Seeking and Processing in Uncertainty Management

Guided by the RISP model (Griffin et al., 1999), the current study examines whether and how exposure to misinformation about COVID-19 prevention motivates or deters effortful seeking and processing of relevant information. The RISP model is one of the most comprehensive models that seeks to understand social psychological motivators of seeking and processing risk information (Yang, Aloe, & Feeley, 2014). In the model, various concepts drawn from the theory of planned behavior (Ajzen, 1991) and other works are depicted as having an indirect impact on information seeking and processing through the model’s central concept of information insufficiency. However, the RISP model does not theorize individuals’ prior exposure to risk information within the model, despite the possible implications of prior exposure on information seeking and processing.

In risk contexts, information seeking and processing are driven by the motivation to reduce uncertainty. Information insufficiency refers to one’s subjective assessment of the gap between their *perceived current knowledge* about a risk and what they feels is sufficient knowledge for adequately coping with the risk (*sufficiency threshold*). Information insufficiency is at the heart of the RISP model because uncertainty reduction occurs only when individuals are sufficiently motivated to engage in the tasks needed to achieve the desired judgmental confidence (Eagly & Chaiken, 1993). While little is known about how exposure to misinformation influences individuals’ perceived (needed) knowledge, misinformation on COVID-19 can potentially make individuals feel overwhelmed with different and inconsistent recommendations on what prevents and cures the disease (Pentina & Tarafdar, 2014). In turn, this sense of being overloaded with information may manifest as having sufficient information on a given issue (i.e., a lower information insufficiency). Indeed, Pentina and Tarafdar (2014) have argued that the amount and variety of unverified information circulating online can make individuals feel overloaded with information, given individuals’ limited capacity to process. Researchers have also suggested that the ambiguity, low quality, and novelty of information can trigger the feeling of overload as these attributes make it more difficult for individuals to process information (Keller & Staelin, 1987; Schneider, 1987). Thus, we posit our first hypothesis on information insufficiency as follows:

**Hypothesis 1 (H1):** Exposure to misinformation will be negatively associated with information insufficiency.

The RISP model theorizes information insufficiency as the primary component that predicts subsequent information seeking and avoidance as well as how the information will be processed. *Information seeking* is defined as a volitional process of acquiring desired information from relevant sources, whereas *information avoidance* refers to deliberately shunning or delaying the acquisition of available information. We treated information seeking and avoidance as two orthogonal constructs, instead of the opposites on a continuum, as they can coexist under some circumstances that involve uncertainty (Yang & Kahlor, 2013). Informed by the heuristic-systematic model (Eagly & Chaiken, 1993), the RISP model posits a dual system of information processing. The dual system includes one that requires more effortful and deeper processing (*systematic processing*) and another that involves more superficial processing and poses fewer cognitive demands on individuals (*heuristic processing*). Griffin et al. (1999) predicted that the drive to overcome information insufficiency motivates individuals to seek more risk-related information and to systematically process the information, while making it less likely for them to heuristically process. Some empirical work has supported the insufficiency principle in this role (Griffin et al., 2002; Kahlor, 2007), while a meta-analysis of the RISP model found limited evidence (Yang, Aloe, & Feeley, 2014).

The conflicting evidence points to the possibility of information insufficiency serving as a mediator between misinformation exposure and information seeking and processing. Prior exposure also may have direct associations with information seeking and processing. Past information exposure and related attitudes have been related to other information behaviors, including information avoidance and information sharing (Kahlor et al., 2016; Yang & Kahlor, 2013; Yang, Kahlor, & Griffin, 2014). Furthermore, a study by Kalichman et al. (2006) suggested a positive relationship between exposure to misinformation about AIDS/HIV and information avoidance behaviors. Thus, we posit the following direct and indirect effects of misinformation exposure on information seeking and avoidance, as well as systematic and heuristic processing.

**Hypothesis 2 (H2):** Exposure to misinformation will be associated with (a) reduced information seeking, (b) increased information avoidance, (c) reduced systematic processing, and (d) increased heuristic processing.
**Hypothesis 3 (H3):** Informational insufficiency will mediate the effect of misinformation on (a) information seeking, (b) information avoidance, (c) systematic processing and (d) heuristic processing.

The RISP model also addresses several psychosocial factors that predict information insufficiency. The most powerful predictor of risk information seeking to emerge from the RISP research is *informational subjective norms*, that is, perceived pressure from others to engage in a given information behavior (Kahlor, 2010; Yang, Aloe, & Feeley, 2014). These norms also constitute an important predictor of information insufficiency (Kahlor, 2010; Yang, Aloe, & Feeley, 2014). Another important RISP concept is *risk perception*, which comprises subjective probability and perceived severity of harm and is the most commonly examined cognitive component of how individuals assess a given risk (Yang, Aloe, & Feeley, 2014). The RISP model also takes into account *affective responses to risk*, such as anxiety and fear, which serve as important heuristic cues in making risk decisions (Finucane et al., 2000). Affective responses, which result from risk perceptions, increase an individual’s desire for information (Griffin et al., 2008; So et al., 2019; Yang & Kahlor, 2013).

One interesting question is whether misinformation exposure influences these psychosocial factors, which would subsequently affect information insufficiency as well as information seeking and processing. There are two different possibilities. If misinformation exposure increases perceived risk, affective response to risk, and informational subjective norms, then this also would increase information insufficiency, thus counterbalancing the negative implications of misinformation hypothesized in H1. In contrast, if misinformation decreases these psychosocial factors, this would further explain H1. To examine these possibilities, we pose the following research question:

**Research Question 1 (RQ1):** What is the role of risk perception, affective response, and informational subjective norms in the relationship between misinformation exposure and information insufficiency?

## Cultural Difference in the Effects of Misinformation

There is limited understanding of why certain individuals or societies are more or less vulnerable to misinformation (Wang et al., 2019). Researchers suggest that older adults (Mitchell et al., 2003), those with lower cognitive ability (De keersmaecker & Roets, 2017), and those who are less educated (Kalichman et al., 2006) are more likely to be misinformed than those who are younger, have higher cognitive ability, or are more educated. Prior research also points to ideological asymmetries in sharing and believing misinformation. The research suggests that people who prioritize conformity and tradition (i.e., conservatives) also tend to emphasize uncertainty reduction, and thus exaggerate within-group consensus and maintenance of homogenous social relationships, both of which contribute to the spread of misinformation (Jost et al., 2018).

Beyond these individual-level characteristics, we lack data comparing the relative susceptibility to misinformation between populations and societies based on cultural differences. Research on cultural differences suggests that uncertainty avoidance, which refers to the “extent to which the members of a culture feel threatened by uncertain or unknown situations” (Hofstede, 1991, p. 113), is a cultural dimension related to anxiety, security needs, and rule orientation. High–uncertainty avoidance cultures tend to be less tolerant about ambiguity and diversity than low–uncertainty avoidance cultures. Because misinformation on COVID-19 prevention is characterized by scientific uncertainty, we suggest that cultural differences in uncertainty avoidance may moderate the effect of misinformation exposure on information seeking and processing.

Moreover, cultural differences in uncertainty avoidance also may change the relative strength of the relationship between information insufficiency and information seeking and processing. That is, those in high–uncertainty avoidance cultures may be more likely to act on their information insufficiency to seek out and effortfully process relevant information in order to reduce their uncertainty, than those in low–uncertainty avoidance cultures. Consistent with this prediction, in the context of climate change, one cross-cultural study based on the RISP model found the information insufficiency– information seeking intention association to be stronger in the U.S. sample (a relatively higher uncertainty avoidance culture) compared to the China sample (a low–uncertainty avoidance culture; Yang, Kahlor, & Li, 2014). Given the conceptual importance of information insufficiency in the RISP model, we extend prior work by comparing the relative strength of the effect of information insufficiency between the U.S. sample and two other countries, one with a higher uncertainty avoidance culture (South Korea, index score = 85) and the other with a lower uncertainty avoidance culture (Singapore, index score = 8) compared to the United States (index score = 46; Hofstede, 1983; Hofstede et al., 2010).

**Research Question 2 (RQ2):** Do the direct and indirect effects of misinformation exposure on information seeking, avoidance, and processing differ between the United States and South Korea or Singapore?

## Method

An online survey was conducted in the early stages of the COVID-19 pandemic^1^ in three countries, the United States (March 16-23, 2020), South Korea (February 24-March 3, 2020) and Singapore (February 25-March 10, 2020). Panel members were recruited from online panel companies: Global Research in South Korea (*N* = 1,500) and Qualtrics in Singapore (*N* = 1,023) and the United States (*N* = 419). We employed quota sampling in terms of age, gender, and ethnicity to match with the national profile of Singapore, South Korea, and the United States. The survey took about 15 minutes to complete and was administered in English in Singapore and United States and in Korean in South Korea. The English survey questionnaire was translated into Korean by two bilingual researchers.

For the combined samples, respondents ranged in age from 18 to 90 (*M* = 42.29, *SD* = 12.62) and consisted of 48.8 % females. The median educational attainment was “some college or an associate’s (2-year) degree.” The majority of the Singapore sample was ethnic Chinese (80.4%), followed by 10.5% Malay. In the U.S. sample, 74.9% self-identified as White and 15.4% identified as Black or African American. South Korea is a monoethnic country. Table 1 presents the sample profile and descriptive statistics by country.

**Table 1.** Sample Profile and Descriptive Statistics.

Factors	United States (*N* = 419), *M* (*SD*) or %	Singapore (*N* = 1,023), *M* (*SD*) or %	South Korea (*N* = 1,500), *M* (*SD*) or %
Age, years	45.90 (16.81)	43.79 (12.44)	40.25 (10.90)
Gender (male), %	49.4	51.7	51.3
Education (High school or less education), %	18.9	15.2	22.1
Some college	36.8	29.1	66.7
University or bachelor’s degree	27.9	44.8	1.6
Graduate degree	16.5	10.9	9.5
Had respiratory diseases in the past few weeks, %	13.8	1.2	5.5
Had cases of COVID-19 in the residing city, %	79.2	100^a^	78.2
Information seeking	3.63 (0.94)	3.27 (0.83)	3.35 (0.73)
Information avoidance	2.33 (1.06)	1.82 (0.76)	2.16 (0.84)
Systematic processing	3.95 (0.81)	3.53 (0.78)	3.57 (0.66)
Heuristic processing	2.96 (0.98)	3.01 (0.72)	3.15 (0.69)
Information insufficiency	39.07 (24.68)	40.25 (15.56)	40.84 (16.40)
Exposure to misinformation	1.63 (0.96)	1.26 (0.52)	1.48 (0.66)
Exposure to general information	3.05 (1.02)	3.22 (0.71)	3.08 (0.64)
Risk perception	200.88 (149.22)	178.30 (118.69)	193.97 (121.93)
Affective responses	3.01 (1.10)	3.00 (0.91)	3.97 (0.73)
Informational subjective norms	3.82 (0.94)	3.11 (1.01)	3.35 (0.77)

a Singapore is a city-state country.

### Measures

#### Information Seeking and Avoidance

Information seeking was measured by five items derived from J.-N. Kim et al. (2012) and J.-N. Kim and Grunig (2011). Sample items, on a 5-point Likert-type scale (1 = *strongly disagree*, 5 = *strongly agree*), included “I regularly check to see if there is any new information about this problem” and “I spend a lot of time learning about this issue” (*M* = 3.36, *SD* = .81, α = .82). Information avoidance was measured by five items adapted from Howell and Shepperd (2016) and Miles et al. (2008). On the same Likert-type scale, items included “I don’t want any more information about COVID-19” and “I avoid learning about COVID-19” (*M* = 2.06, *SD* = 0.87, α = .88).

#### Systematic and Heuristic Processing

Systematic processing was assessed by three items derived from Yang et al. (2010) and Yang et al. (2019). On a 5-point scale (1 = *not at all*, 5 = *very much*), sample items included “After I encounter information about COVID-19, I stop and think about it” and “For me to understand about COVID-19, the more viewpoints I get the better” (*M* = 3.61, *SD* =0 .74, α = .73). Heuristic processing was assessed by three items from Yang et al. (2010) and Kahlor et al. (2003). Using the same 5-point scale, items included, for example, “When I come across information about COVID-19, I focus on only a few key points” (*M* = 3.07, *SD* = .75, α = .64). Although Cronbach’s alphas for these scales are relatively weak, they are comparable to the one used by Yang et al. (2010), who suggest that these processing scales are still under development and thus have room for improvement (also see Deng & Chan, 2017, as Yang et al., 2010, reported omega rather than alpha).

#### Information Insufficiency

To calculate information insufficiency, we separately assessed perceived current knowledge and sufficiency threshold (Griffin et al., 2008; Yang et al., 2019). To assess perceived current knowledge, participants rated to what extent they currently know about COVID-19 on a scale of 0 (*knowing nothing*) to 100 (*knowing everything; M* = 65.24, *SD* = 19.39). For sufficiency threshold, participants estimated how much knowledge they would need in order to deal adequately with the risk of COVID-19 on a scale of 0 (*need to know nothing*) to 100 (*need to know everything you could possibly know; M* = 73.00, *SD* = 20.03). We employed the analysis of partial variance (Cohen & Cohen, 1983) to compute information insufficiency^2^ (*M* = 40.38, *SD* = 17.55) that contains the residual variance of information threshold accounting for the variance of perceived current knowledge (Rosenthal, 2013). This approach helps to address the limitations of using a raw difference score (e.g., sensitive to floor and ceiling effects) and the regressed change approach (e.g., the inflated explained variance in information insufficiency).

#### Exposure to COVID-19 Information

Exposure to misinformation was assessed with five claims on COVID-19 prevention measures that were identified as false at the time of data collection^3^ (WHO, 2020): (a) gargling with mouthwash, (b) eating garlic, (c) avoiding pets, (d) vaccination against pneumonia, and (e) regularly rinsing the nose with saline. On a 4-point scale (1 = *not at all*, 4 = *a lot of times*), participants reported how often they had heard that each of the five claims from eight different information sources (e.g., news app or website, social media app or website, medical or health websites, television and radio news; Tan et al., 2015). Cronbach’s α for the exposure scales across the five claims ranged from .96 to .97, and items were averaged to create composite scores. The composite scores were further averaged into an index of exposure to misinformation (*M* = 1.43, *SD* = 0.68, α = .95).

For comparison purpose, we also examined exposure to general COVID-19 information without specifying the information content. On a 5-point scale (1 = *never*, 5 = *very often*), participants reported how often they learned about COVID-19 using 10 different information sources (Rains, 2007; e.g., websites or social networking site [SNS] of governmental health agencies, print or online newspapers, SNS of newspapers, individual SNS, television). Responses were averaged into a score of exposure to general COVID-19 information (*M* = 3.13, *SD* = .73, α = .95). Exposure to misinformation and exposure to general COVID-19 information were moderately correlated (*r* = .43, *p* < .001).

#### Risk Perception

Two components of risk perception were assessed: perceived susceptibility and severity. To assess perceived susceptibility (Brewer et al., 2004), participants estimated their chances of contracting COVID-19 in several weeks if they do not take any preventive actions on a given slider between 0% and 100% at 10% intervals (*M* = 52.25, *SD* = 28.24). On a 5-point Likert-type scale (1 = *strongly disagree*, 5 = *strongly agree*), we used three items derived from Weinstein (2000) to measure perceived severity (e.g., “I think that COVID-19 is a very dangerous disease”; *M* = 3.50, *SD* = 0.98, α = .86). Based on convention (Griffin et al., 2008; Weinstein, 2000), we multiplied perceived susceptibility and severity to create an index of risk perception (*M* = 189.51, *SD* = 125.34).

#### Affective Responses

We assessed negative affective responses experienced during the COVID-19 pandemic such fear, anger, sadness, and anxiety derived from prior work (Yang, 2016; Yang et al., 2019). These emotions have been reported to be frequently experienced in crises and pandemic situations (Jin et al., 2012; H. K. Kim & Niederdeppe, 2013). On a 5-point scale (1 = *not at all*, 5 = *very much*), participants rated their feelings toward the COVID-19 situation on the following emotions themed under fear (afraid, fearful, scared), anger (angry, mad, irritated), sadness (sad, downhearted, unhappy), and anxiety (anxious, worried, concerned). Responses were averaged to create a scale of affective responses (*M* = 3.49, *SD* = 0.98, α = .95).

#### Informational Subjective Norms

Derived from Yang and Kahlor (2013), we used four items asking participants’ perception of other’s expectations about their seeking COVID-19–related information on a 5-point scale (1 = *not at all*, 5 = *very much*; e.g., “Most people who are important to me think that I should seek information about COVID-19”). Responses were averaged to create a scale of informational subjective norms (*M* = 3.33, *SD* = 0.91, α = .88).

### Analytic Approach

**Figure 1.**
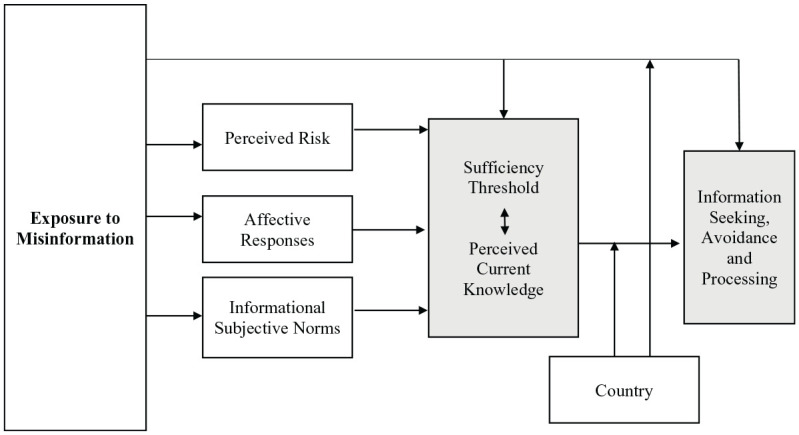
Informational implications of misinformation on COVID-19.

To examine our hypotheses and research questions (summarized in Figure 1), we used hierarchical ordinary least squares regression, which allowed us to enter variables in separate blocks to test the incremental assessment of *R*^2^ in each step as well as the relative effects of variables while accounting for those entered together or in earlier steps (Cohen et al., 2003). All the analyses controlled for demographic factors (i.e., age, gender, education, country), having a respiratory disease in the past few weeks (yes/no), and presence of local cases of COVID-19 in the subject’s city (yes/no). Multicollinearity tests showed tolerance values above zero and variance inflation factor values below the conventional cutoff value of 10 for all variables entered in the models (Cohen et al., 2003).

We entered exposure to misinformation and general information on COVID-19 in the first block along with other control factors (testing H1 and H2), and RISP model components (risk perception, affective response, informational subjective norms, and information insufficiency) in the second block. Addressing H3 and RQ1, we tested a serial mediation model (Model 80) with PROCESS macro (Hayes, 2017) to investigate the indirect effects of misinformation on information seeking, avoidance, and processing, separately mediated through risk perception, affective responses, and information subjective norms, as well as serially via information insufficiency. Addressing RQ2, the conditional indirect effects via information insufficiency by country were analyzed with PROCESS macro Model 15. The U.S. sample served as a reference group given its middle position in regard to the level of uncertainty avoidance (Hofstede, 1983). We estimated confidence intervals (CIs) with 5,000 bootstrap samples.

## Results

### Effects of Misinformation

We hypothesized that exposure to misinformation would be negatively associated with information insufficiency (H1). As shown in Table 2, H1 was supported. Information insufficiency was negatively correlated with misinformation exposure (β = -0.12, *p* < .001) and positively with general information on COVID-19 (β = 0.11, *p* < .001). Risk perception (β = 0.13), affective response (β = 0.14), and information subjective norms (β = 0.09) were also positively associated with information insufficiency (all *p* < .001).

**Table 2.** Ordinary Least Squares Regression Predicting Risk Perception, Affective Response, Informational Subjective Norms, and Information Insufficiency.

	Risk perception		Affective response		Informational subjective norms		Information insufficiency	
Predictors	β	*t*	β	*t*	β	*t*	β	*t*
Step 1								
Age	0.086	4.68^***^	0.001	0.02	0.077	4.82^***^	0.106	5.68^***^
Female (vs. male)	0.059	3.25^**^	0.069	4.42^***^	−0.022	−1.38	0.073	3.97^***^
Education	−0.042	−2.25^*^	−0.017	−1.03	0.026	1.55	−0.027	−1.40
Country (reference = United States)	—	—	—	—	—	—	—	—
Singapore	−0.077	−2.72^**^	−0.035	−1.44	−0.421	−16.91^***^	−0.004	−0.14
South Korea	−0.001	−0.04	0.481	20.20^***^	−0.242	−9.92^***^	0.052	1.83
Respiratory disease	0.083	4.48^***^	−0.010	−0.65	−0.039	−2.39^*^	−0.009	−0.48
Local cases	0.050	2.65^**^	0.038	2.33^*^	0.03	1.85	0.005	0.24
Misinformation	0.042	1.98^*^	0.045	2.48^*^	0.038	2.08^*^	−0.118	−5.55^***^
General information	0.171	8.38^***^	0.241	13.84^***^	0.451	25.26^***^	0.11	5.29^***^
Step 2								
Risk perception							0.126	6.36^***^
Affective response							0.141	6.02^***^
Informational subjective norms							0.094	4.41***
	Adjusted *R*^2^ = .058, *F*(9, 2932) = 21.20^***^		Adjusted *R*^2^ = .314, *F*(9, 2932) = 150.43^***^		Adjusted *R*^2^ = .280, *F*(9, 2932) = 128.01^***^		Adjusted *R*^2^ = .087, Δ*R*^2^= .052^***^, *F*(12, 2929) = 23.12^***^	

* *p* < .05. ^**^p < .01. ^***^p < .001.

We also predicted that exposure to misinformation would be negatively associated with information seeking and systematic processing, and positively associated with information avoidance and heuristic processing (H2a-d). As shown in Table 3, H2a was not supported, as misinformation was positively associated with information seeking (β = 0.045, *p* = .006). However, H2b and H2d were supported, as misinformation was positively associated with information avoidance (β = 0.373, *p* < .001) and with heuristic processing (β = 0.273, *p* < .001). As predicted, H2c also was supported, as systematic processing was negatively associated with misinformation exposure (β = −0.048, *p* = .012). At Step 2, controlling for other RISP components, information insufficiency was negatively associated with information avoidance (β = −0.104, *p* < .001) and heuristic processing (β = −0.067, *p* = .002), whereas it was positively associated with systematic processing (β = 0.104, *p* < .001). No association was found with information seeking (β = 0.022, *p* = .096).

**Table 3.** Ordinary Least Squares Regression Predicting Risk Information Seeking, Avoidance, and Processing.

	Information seeking		Information avoidance		Systematic processing		Heuristic processing	
Predictors	β	*t*	β	*t*	β	*t*	β	*t*
Step 1								
Age	−0.028	−1.94	−0.076	−4.39^***^	0.05	2.97^**^	−0.068	−3.75^***^
Female (vs. male)	0.034	2.40^*^	−0.059	−3.48^**^	0.017	1.02	−0.006	−0.36
Education	0.026	1.76	−0.038	−2.17^*^	0.003	0.15	−0.041	−2.23^*^
Country (reference = United States)	—	—	—	—	—	—	—	—
Singapore	−0.289	−13.08^***^	−0.145	−5.45^***^	−0.346	−13.35^***^	0.095	3.41^**^
South Korea	−0.186	−8.61^***^	−0.071	−2.71^**^	−0.266	−10.49^***^	0.126	4.61^***^
Respiratory disease	−0.029	−2.03^*^	0.056	3.25^**^	−0.013	−0.77	0.008	0.42
Local cases	0.031	2.10^*^	−0.032	−1.79	0.035	2.05^*^	−0.012	−0.66
Misinformation	0.045	2.75^**^	0.373	18.95^***^	−0.048	−2.52^*^	0.273	13.21^***^
General information	0.62	39.18^***^	−0.181	−9.47^***^	0.451	24.26^***^	0.015	0.76
Step 2								
Risk perception	0.033	2.34^*^	−0.038	−2.07^*^	0.037	2.16^*^	−0.051	−2.60^*^
Affective responses	0.11	6.53^***^	−0.041	−1.87	0.098	4.86^***^	0.022	0.93
Informational subjective norms	0.312	20.37^***^	−0.104	−5.22^***^	0.302	16.50^***^	−0.067	−3.18^**^
Information insufficiency	0.022	1.67	−0.12	−6.96^***^	0.104	6.60^***^	−0.061	−3.36^**^
	Adjusted *R*^2^ = .530, Δ*R*^2^= .097, *F*(13, 2928) = 256.48^***^		Adjusted *R*^2^ = .207, Δ*R*^2^= .033^***^, *F*(13, 2928) = 60.11^***^		Adjusted *R*^2^ = .327, Δ*R*^2^ = .108^***^, *F*(13, 2928) = 111.05^***^		Adjusted *R*^2^ = .102, Δ*R*^2^= .011^***^, *F*(13, 2928) = 26.72^***^	

* *p* < .05. ^**^p < .01. ^***^p < .001.

### Indirect Effects of Misinformation

We predicted indirect effects of misinformation on information seeking, avoidance, and processing via informational insufficiency (H3). In light of the predictions of the RISP model, we also included risk perception, affective responses, and informational subjective norms in a serial mediation model using PROCESS macro (Model 80; Table 4). Our results supported H3b, H3c, and H3d. As predicted, the indirect effect via information insufficiency was significant on information avoidance (95% CI [.012, .030]), systematic processing (CI [−.023, −.009]), and heuristic processing (CI [.003, .016]). However, no indirect effect was found on information seeking (CI [−.008, .001]); thus, H3a was not supported.

**Table 4.** Mediation of Misinformation Effect on Information Seeking, Avoidance, and Processing.

Mediators	Information behaviors and processing			
	Information seeking	Information avoidance	Systematic processing	Heuristic processing
Risk perception	.002 [−.001, .005]	−.002 [−.006, .001]	.002 [−.0003, .005]	−.002 [−.007, .0002]
Affective responses	**.006 [.001, .012]**	−.002 [−.007, .001]	**.005 [.0005, .010]**	.001 [−.002, .004]
Informational subjective norms	**.014 [.002, .026]**	−**.005 [**−**.010,** −**.001]**	**.013 [.002, .024]**	−**.003 [**−**.007,** −**.0002]**
Information insufficiency	−.004 [−.008, .001]	**.02 [.012, .030]**	−**.015 [**−**.023,** −**.009]**	**.009 [.003, .016]**
Risk perception → Information insufficiency	.0001 [−.0001, .0005]	−.001 [−.002, .0001]	.0006 [−.0001, .001]	−.0004 [−.001, .0000]
Affective responses → Information insufficiency	.0002 [−.0001, .0005]	−**.001 [**−**.002,** −**.0001]**	**.0007 [.0001, .0016]**	−.0004 [−.001, .0000]
Informational subjective norms → Information insufficiency	**.0001 [.0000, .0003]**	−**.0005[**−**.001,** −**.0001]**	**.0004 [.0001, .001]**	−.0002 [−.0006, .0000]
Direct effect	.034 [−.001, .069]	**.466 [.418, .515]**	−**.058 [**−**.096,** −**.020]**	**.297 [.252, .342]**
Total indirect effect	**.018 [.003, .035]**	.009 [−.004, .021]	.005 [−.010, .022]	.004 [−.005, .012]

*Note*. PROCESS Model 80; unstandardized point estimate of indirect effects [bias-corrected 95% confidence interval] with 5,000 bootstrap samples. Controlled for demographic factors, relevant hazard experience, and general information exposure. Significant effects in bold text.

As for the serial mediation, addressing RQ1, information insufficiency serially mediated the effect of misinformation on information avoidance and systematic processing separately via affective responses (95% CI [−.002, −.0001]; CI [.0001, .0016], respectively) and information subjective norms (CI [−.001, −.0001]; CI [.0001, .001], respectively), but not via risk perception. On information seeking, only the informational subjective norms – information insufficiency path appeared to serially mediate the effect of misinformation (95% CI [.0000, .0003]). The total indirect effect was significant only on information seeking (CI [−.001, −.0001]), whereas the direct effect of misinformation was significant across information avoidance (CI [.418, .515]), systematic processing (CI [−.096, −.02]), and heuristic processing (CI [.252, .342]).

### Conditional Direct and Indirect Effects of Misinformation

**Figure 2.**
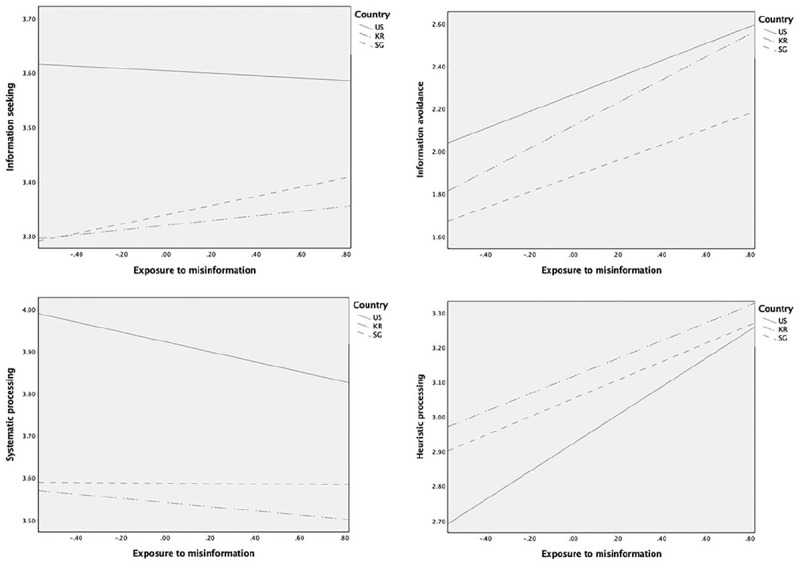
Effects of misinformation on information seeking, avoidance, and processing by country. *Note*. US = United States; KR = South Korea; SG = Singapore.

**Figure 3.**
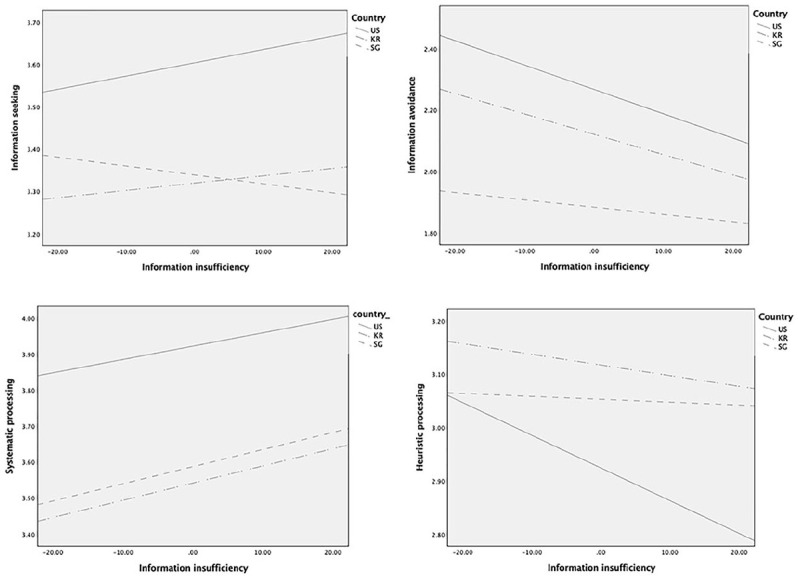
Effects of information insufficiency on information seeking, avoidance, and processing by country. *Note*. US = United States; KR = South Korea; SG = Singapore.

Finally, we sought to explore whether the direct and indirect effects of misinformation are further moderated by country (RQ2). We used PROCESS macro (Model 15; Table 5) to examine the moderated mediation via information insufficiency by country (U.S. sample served as a reference group) on information seeking, avoidance, and processing. Figure 2 presents the effects of misinformation exposure by country, and Figure 3 presents the effects of information insufficiency by country.

**Table 5.** Conditional Effects and Moderated Mediation by Country.

	Information behaviors and processing			
	Information seeking	Information avoidance	Systematic processing	Heuristic processing
Conditional direct effects of misinformation				
United States	−.022 [−.082, .038]	**.399 [.315, .486]**	−**.118 [**−**.184,** −**.052]**	**.408 [.330, .486]**
Singapore	**.085 [.018, .153]**	**.370 [.276, .465]**	−.004 [−.078, .070]	**.265 [.178, .352]**
South Korea	.044 [−.002, .089]	**.538 [.474, .602]**	−.050 [−.100, .001]	**.256 [.197, .315]**
Conditional indirect effects via info insufficiency				
United States	−**.011 [**−**.020,** −**.003]**	**.029 [.014, .046]**	−**.013 [**−**.026,** −**.002]**	**.022 [.009, .037]**
Singapore	.008 [−.003, .019]	.009 [−.002, .020]	−**.017 [**−**.030,** −**.007]**	.002 [−.011, .015]
South Korea	−.006 [−.014, .0001]	**.024 [.013, .037]**	−**.017 [**−**.027,** −**.009]**	.007 [−.001, .016]
Index of moderated mediation				
Singapore (vs. United States) conditional indirect effects	**.019 [.006, .034]**	−**.020 [**−**.040,** −**.003]**	−.004 [−.020, .011]	−**.020 [**−**.041,** −**.003]**
South Korea (vs. United States) conditional indirect effects	.005 [−.005, .016]	−.005 [−.022, .012]	−.004 [−.019, .009]	−**.015 [**−**.032,** −**.001]**

*Note*. PROCESS Model 15; unstandardized point estimate of indirect effects [bias-corrected 95% confidence interval] with 5,000 bootstrap samples. Controlled for demographic factors, relevant hazard experience, general information exposure, risk perception, affective response, and informational subjective norm. Significant effects in bold text. United States served as a reference.

In a model predicting information seeking, the direct effect of misinformation differed between Singapore and United States (*p* = .015; South Korea-United States comparison, *p* = .07) such that it was significant only in the Singapore sample (Β = .085, *p* = .013; Β_*US*_ = −.022, *p* = .47; Β_*KR*_ = .044, *p* = .06). In contrast, the effect of information insufficiency on information seeking was significant only in the US sample (Β = .003, *p* = .005), which was significantly different from that of the Singapore sample (*p* < .001; Β_*SG*_ = −.002, *p* = .06) but not from the South Korea sample (*p* = .32; Β_*KR*_ = .002, *p* = .054). The conditional indirect effect was significant only in the US sample (95% CI [−.020, −.003]) and this effect statistically differed only from the Singapore sample (index of moderated mediation = .019, CI [.006, .034]).

In predicting information avoidance, the direct effect of misinformation was significant across all three countries (Β_*US*_ = .399, Β_*SG*_ = .370, Β_*KR*_ = .538, all *p* < .001), but the effect size significantly differed only between the U.S. and South Korea samples (*p* = .006; United States-Singapore comparison, *p* = .63). The effect of information insufficiency on information avoidance was significant in the U.S. and South Korea samples (Β_*US*_ = −.008, Β_*KR*_ = −.007, all *p* < .001) but not in the Singapore sample (Β_*SG*_ = −.002, *p* = .13); thus, only the contrast between the United States and Singapore was significant (*p* = .011). The conditional indirect effect was significant only in the U.S. (95% CI [.014, .046]) and South Korea samples (CI [.013, .37]); there was a significant moderated mediation for the United States-Singapore contrast (index = −.020, CI [−.040, −.003]).

As for systematic processing, the direct effect of misinformation was significant only for the U.S. sample (Β_*US*_ = −.118, *p* < .001; Β_*SG*_ = −.004, *p* = .91; Β_*KR*_ = −.050, *p* = .053), and it contrasted significantly with the effect in the Singapore sample (*p* = .02) but not the South Korea sample (*p* = .09). The effect of information insufficiency did not differ by country (United States-South Korea comparison, *p* = .49; United States-Singapore comparison, *p* = .54) and all conditional indirect effects were significant on systematic processing regardless of country (95% CI United States [−.026, −.002]; Singapore [−.030, −.007]; South Korea [−.027, −.009]), suggesting no significant moderated mediation.

In predicting heuristic processing, the direct effect of misinformation was significant across all countries (Β_*US*_ = .408, Β_*SG*_ = .265, Β_*KR*_ = .256, all *p* < .001), while the effect size being stronger in the U.S. sample than in the Singapore (*p* = .012) or South Korea samples (*p* = .001). The effect of information insufficiency was significant only in the U.S. sample (Β_*US*_ = −.006, *p* < .001; Β_*SG*_ = −.001, *p* = .71; Β_*KR*_ = −.002, *p* = .09), and its coefficient was significantly different from that of the Singapore (*p* = .006) and South Korea samples (*p =* .023). Accordingly, the conditional indirect effect was significant only in the U.S. sample (95% CI [.010, .037]) and the moderated mediation was significant for both United States-Singapore (index = −.02, CI [−.041, −.003]) and United States-South Korea comparisons (index = −.015, CI [−.032, −.001]).

## Discussion

The COVID-19 pandemic represents one of the biggest challenges to global human well-being to date. An epidemic of misinformation makes this formidable challenge even more so by impeding people from getting correct information on how to prevent and curb the spread of the disease. Based on a multicountry survey conducted in the early stages of the global pandemic, this study documents that exposure to misinformation demotivates individuals from seeking out and thoughtfully processing information on COVID-19. Our intercountry comparisons suggest, however, that the influence of misinformation exposure may not be equivalent across different populations and cultures. Thus, we provide important insights for theory building, as well as for the mitigation of misinformation effects across populations as they all face a common goal—to prevent and curb the spread of disease.

This study found that exposure to misinformation was negatively associated with information insufficiency. That is, when people encounter misinformation, they perceived less informational need for adequately preventing and treating COVID-19. It is noteworthy that exposure to misinformation reduced both sufficiency threshold and current knowledge, when these variables were analyzed separately. Yet the decrease in sufficiency threshold was greater than that of current knowledge, which resulted in lower information insufficiency. Unlike the association of misinformation, exposure to general information was positively associated with information insufficiency. This suggests that the influence of misinformation is distinguished from that of general information on COVID-19. In the early stages of a novel disease pandemic, exposure to general information on the unknown risk at hand may make individuals realize that they need more information, whereas the opposite is true for misinformation.

Information insufficiency served as a significant mediator of the relationship between misinformation exposure and information avoidance, systematic processing, and heuristic processing. That is, when individuals perceived that they know enough about COVID-19 as a result of misinformation exposure, they were more likely to avoid information and heuristically process (rather than systematically process) relevant information. This counters the findings from a study that assessed *perceived* misinformation exposure (Hameleers et al., 2020). As Vraga and Bode (2020) addressed, the public may have different perceptions than what is agreed upon among experts (e.g., WHO) on misinformation, thus having differential implications on information behaviors. On the other hand, when other mediators suggested in the RISP model are taken into account, the total indirect effects were not significant on these information outcomes. Notably, affective response and informational subjective norms, both of which could be triggered by exposure to misinformation, appear to counterbalance the mediating role of information insufficiency. While information insufficiency was not directly linked to information seeking, misinformation exposure was indirectly associated with information seeking when mediated by affective response and informational subjective norms.

Across all three countries, exposure to misinformation had a significant direct association with information avoidance and heuristic processing. This relationship was stronger in the South Korea sample for information avoidance (vs. United States) and in the U.S. sample for heuristic processing (vs. South Korea and Singapore). It is also noteworthy that exposure to misinformation had a direct relationship with information seeking only in the Singapore sample, and with systematic processing only in the U.S. sample. These results suggest that exposure to misinformation may have different implications on information seeking or processing depending on culture or population. That is, misinformation exposure may have more implications on how Americans process information, whereas for South Koreans or Singaporeans it will be reflected in their information seeking or avoidance. These may reflect cultural differences in how people manage uncertainties or contextual factors such as partisanship and information sources. In particular, the stronger relationship between misinformation and information avoidance in the South Korea sample (vs. United States) may reflect the high–uncertainty avoidance culture of South Korea (Hofstede et al., 2010). In light of the direct positive association with both seeking and avoidance in the Singapore sample, Singaporeans may be uniquely motivated to deal with misinformation having ambivalent responses toward such information.

Interestingly, only in the U.S. sample, information insufficiency served as a constant predictor across all information outcomes. In the South Korea sample, information insufficiency was significantly associated only with information avoidance and systematic processing. In the Singapore sample, only systematic processing was associated with information insufficiency. Similar to our findings, Yang, Kahlor, and Li (2014) also found a stronger relationship between information insufficiency and information seeking intention in the U.S. sample than the China sample. Collectively, Western populations may be more likely to be influenced by epistemic motivation than Eastern populations, regardless of uncertainty avoidance tendencies. Instead, cultural differences in perceptions of personal control or ability to seek, process, and retain information may be closely related to the differential effects of information insufficiency. Alternatively, given that the RISP model was developed in the Western context, the model and its measurements may better reflect Westerners’ seeking and processing tendencies. Future work should examine these possibilities in cross-cultural contexts.

This study has several limitations to note. First, the sample sizes were not balanced among the three countries examined and these countries were differently affected by COVID-19 at the time when the surveys were conducted. While we controlled for risk characteristics and relevant experience, it was not possible to account for all contextual factors that could confound the results. Nonetheless, we believe that it is imperative to document public sentiment and responses during an actual pandemic, and thus this work could have unique value in studying misinformation effects. Second, this study cannot confirm causal orderings proposed in the conceptual model due to the cross-sectional nature of the data. Future work should consider employing a longitudinal or experimental design to support causal statements about the proposed relationships here. Last, while there are multiple types of COVID-19-related misinformation circulating on the internet, we focused on five false claims relevant to the prevention of COVID-19 that reflect the state of scientific evidence at the time of data collection. It would be beneficial for future studies to investigate additional types of misinformation to better understand misinformation effects.

Despite these limitations, this study makes important contributions to the extant literature on misinformation and information seeking and processing. First, this is the first study that examined implications of misinformation exposure on information seeking and processing. Because information seeking and processing constitute important components in managing uncertainty and risk situations (Griffin et al., 1999), understanding the mechanisms of how misinformation affects these informational behaviors offers crucial insights into human tendencies under uncertainty. Second, this is one of just a few studies to make comparisons across multiple countries that are simultaneously affected by a common risk in studying information seeking and processing. Studies in these areas have often focused on one cultural, mostly Western, context, and intercultural comparisons have been scarce. Comparing the relative predictive utility of a theoretical framework across different cultural contexts and populations is important for theory development.

On the practical front, critical assessment of information as well as active seeking of quality information are crucial for mitigating the false beliefs that could be formed based on misinformation. Given that misinformation demotivates individuals from these important information activities during a disease pandemic, it is necessary to minimize exposure to such incorrect information and to deliver evidence-based health advisories. To this end, risk communicators and government authorities should continuously monitor and clarify emerging misinformation on various online platforms to prevent the public’s misperception and engagement in fake remedies or scientifically unproven measures. In light of the counterbalancing role of informational subjective norm we found, it would be beneficial to emphasize the social expectation on keeping up with health advisories to minimize the adverse effects of misinformation exposure. In dealing with global pandemics, like COVID-19, it would be essential for international and local health agencies to take into account differences in culture in communicating risk. For example, compared to low–uncertainty avoidance cultures (e.g., Singapore, Sweden), high–uncertainty avoidance cultures (e.g., South Korea, Japan, Germany) may be less tolerant about information uncertainty (misinformation) as well as changes in health advisories, which are inevitable in most pandemic situations. In high–uncertainty avoidance culture, clear and consistent risk communications as well as implementation of formal governing structures (e.g., laws) could be particularly beneficial for mitigating uncertainty.

## Notes

1 During data collection, local cases of COVID-19 increased from 4,604 to 44,189 in the United States (out of 328.2 million total population), from 833 to 5,186 in South Korea (out of 51.6 million), and from 96 to 166 in Singapore (out of 5.6 million).

2 In keeping with Cohen and Cohen (1983), we used perceived knowledge to predict sufficiency threshold, and noted the unstandardized regression slope (Β). Then, information insufficiency was computed by subtracting perceived knowledge * Β from sufficiency threshold.

3 In March 2020, WHO removed avoiding pets and gargling with mouthwash from myth busters in response to the emerging evidence on these measures. There is no evidence that pets have infected humans with COVID-19, but an infected dog was found in Hong Kong. As well, there is no proof that gargling prevents respiratory infections caused by COVID-19, but health experts note that there is little downside in gargling.
